# Intravitreal tPA Injection and Pneumatic Displacement for Submacular Hemorrhage in a 10-Year-Old Child

**DOI:** 10.1155/2016/9809583

**Published:** 2016-09-18

**Authors:** Takayuki Tsuyama, Hiroshi Hirose, Tomohiro Hattori

**Affiliations:** Department of Ophthalmology, National Hospital Organization Nagoya Medical Center, 4-1-1 Sannomaru, Nakaku, Nagoya, Aichi 460-0001, Japan

## Abstract

*Background*. Submacular hemorrhage can occur after blunt trauma to the eye. Intravitreal tissue plasminogen activator (tPA) and gas injection are often used for treatment and are effective for submacular hemorrhage caused by age-related macular degeneration. This report describes the clinical outcome in a child with submacular hemorrhage caused by traumatic choroidal rupture who underwent successful intravitreal tPA injection and pneumatic displacement.* Case Presentation*. A 10-year-old boy developed sudden decrease of vision and a central scotoma in his right eye after trauma. Submacular hemorrhage was found in the eye. Visual acuity was 20/70 OD. Tissue plasminogen activator (12.5 *μ*g in 0.05 mL) and 0.3 mL of pure sulfur hexafluoride were injected into the vitreous cavity under general anesthesia. After surgery, the patient was instructed to maintain a prone position. Displacement of the submacular hemorrhage from the fovea revealed a choroidal rupture, presumed to be the cause of the hemorrhage. After 4 months of follow-up, visual acuity was restored and final visual acuity is 20/16.* Conclusion*. Intravitreal tPA and gas injection can be an effective treatment for children with submacular hemorrhage.

## 1. Introduction

Submacular hemorrhage can occur after blunt trauma to the eye. In a patient with traumatic submacular hemorrhage, intervention should be considered because the natural history can be associated with poor visual acuity [1, 2]. In 1996, Herriot reported a technique for treating submacular hemorrhage using intravitreal tissue plasminogen activator (tPA) and gas injection [3]. This technique is often used and is effective for submacular hemorrhage caused by age-related macular degeneration [4]. We used this procedure without vitrectomy to good effect in a pediatric patient with traumatic submacular hemorrhage but without posterior vitreous detachment.

## 2. Case Presentation

**Figure 1:**
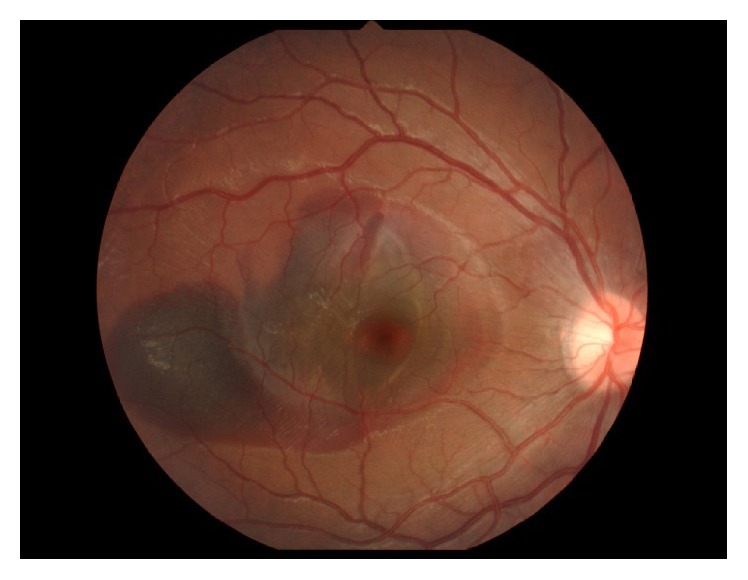
Fundus image showing hemorrhage in the fovea.

**Figure 2:**
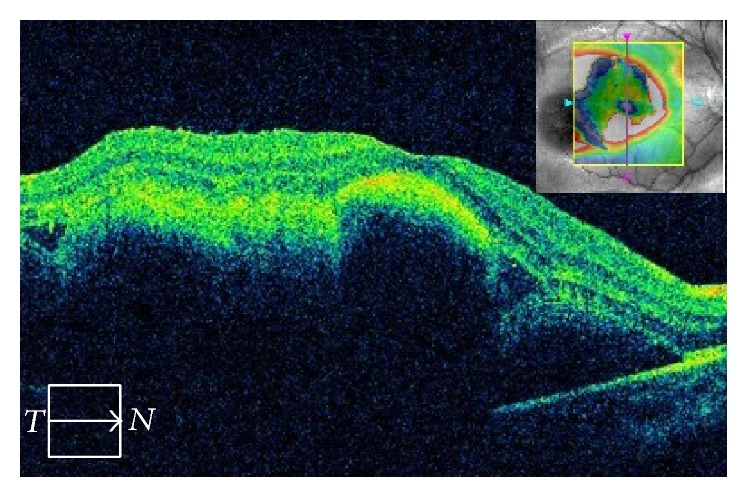
Submacular hemorrhage detected by optical coherence tomography.

**Figure 3:**
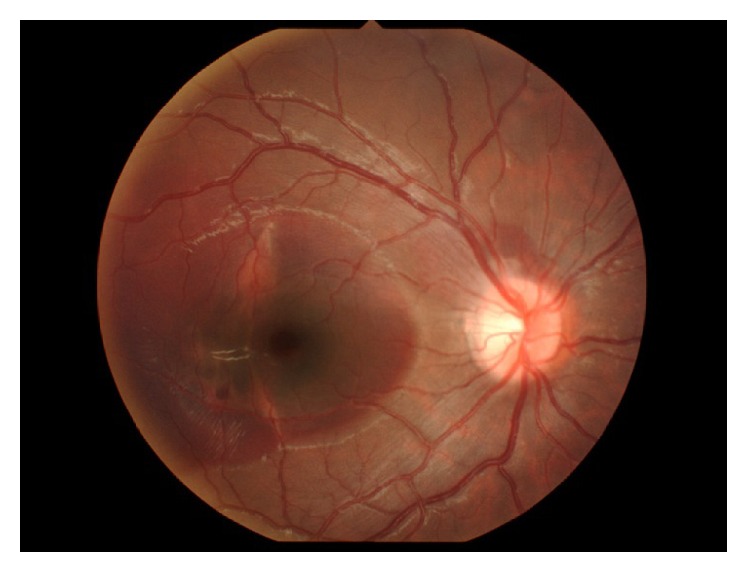
Choroidal rupture observed after displacement.

A 10-year-old boy presented to a local doctor with a sudden decrease of vision and a central scotoma in the right eye two days after being hit in that eye with a wooden stick. A hemorrhage was found in the fundus at that time, so the patient was referred to our hospital, where a hemorrhage was observed in the fovea (Figure 1). Corrected visual acuity was 20/70 and intraocular pressure was 12 mmHg OD. Optical coherence tomography scanning of the right eye showed submacular hemorrhage (Figure 2). We performed paracentesis (0.3 mL) under general anesthesia to reduce the intraocular pressure. Next, we injected tPA (12.5 *μ*g in 0.05 mL) into the vitreous cavity and waited 15 minutes for the hemorrhage to dissolve. We then performed a further paracentesis (0.1 mL), followed by injection of pure sulfur hexafluoride (SF6, 0.3 mL) into the vitreous cavity. After surgery, we instructed the patient to maintain a prone position as much as possible for a week. One day later, the hemorrhage had started to move out of the fovea, revealing a choroidal rupture beneath (Figure 3). We considered this choroidal rupture to be the cause of the hemorrhage.

**Figure 4:**
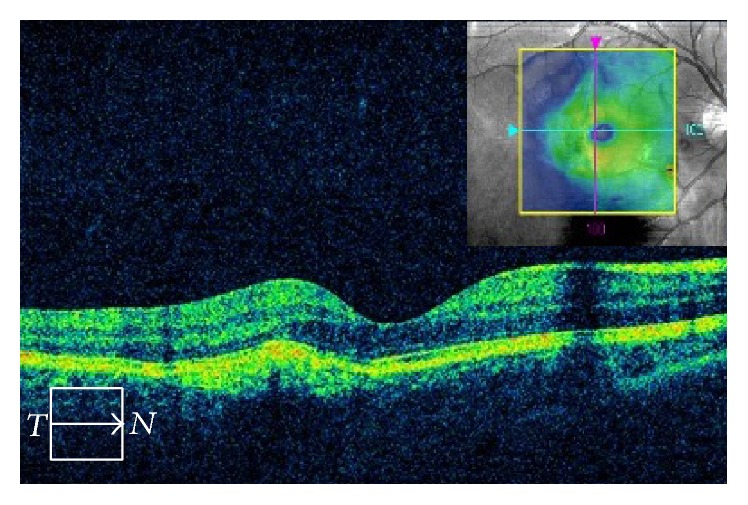
Optical coherence tomography showing intact macula.

The macula was not damaged (Figure 4) and visual acuity improved to 20/16 by four months and 20/13 by seven months after the above treatment. The submacular hemorrhage had been successfully displaced out of the fovea. In spite of the remaining choroidal rupture, the patient has not complained of any symptoms, including metamorphopsia, after 3 years of follow-up.

## 3. Discussion

Approximately 5% of cases of blunt ocular trauma result in rupture of the choroid [5, 6]. Although patients with submacular hemorrhage may have spontaneous visual improvement [7] and photoreceptor function may be reversible [8], most patients with traumatic choroidal rupture do not recover visual acuity to better than 20/40 [9].

The mechanism by which subretinal blood damages the overlying neurosensory retina may be explained as follows. Fibrin clots may dislodge photoreceptors from the retina; iron derived from hemoglobin may have a destructive effect on the outer layers of the retina; and the subretinal hemorrhage may form a direct mechanical barrier preventing the photoreceptors from receiving metabolic support from the retinal pigment epithelium and choriocapillaris [10–12].

In experimental cat models, the immediate damage due to the mechanical shearing of photoreceptors during fibrin clot formation has been reported to occur as early as one hour after induction of subretinal hemorrhage [13, 14]. Severe degeneration of the inner and outer retinal layers occurred within 7–14 days in the holangiotic feline retina [12, 13]. In experimental rabbit models, irreversible retinal damage was observed in less than 24 hours of subretinal injection of blood [11] and significant destruction of the outer retina was observed within 3–7 days in the merangiotic rabbit retina [15]. Therefore, many investigators have advocated the early removal of blood from the fovea to minimize these damaging effects [16]. Delayed evacuation of a submacular hemorrhage (for ≥7 days after onset) has been reported to be a risk factor for a poor surgical outcome [17]. Hemorrhage that does not persist for more than 14 days has been associated with better restoration of vision [18].

In 1996, Herriot introduced a less invasive procedure for treating submacular hemorrhage, that is, intravitreal tPA injection to liquefy the subretinal blood clot and long-acting intravitreal injection of expansile gas in combination with postoperative face-down positioning to displace the subfoveal hemorrhage pneumatically [3]. Although this technique has a high anatomic success rate with no significant complications and has been widely accepted, there are several considerations with regard to the adjuvant effectiveness and potential risk of tPA [19, 20]. Dose-dependent adverse retinal reactions to intravitreal tPA have been documented in experimental animal models and clinical case reports [16]. Johnson et al. observed toxic retinal reactions in rabbit eyes after injection of t-PA at concentrations ≥ 50 *μ*g per 0.1 mL [21]. Lewis et al. found no morphologically toxic effect on the retina in rabbits that received injection of tPA at a dose of 25 *μ*g or 50 *μ*g per 0.1 mL [22]. Hrach et al. observed pigmentary changes in the fundus, reduced B-wave amplitude on the electroretinogram, and loss of photoreceptor elements with necrosis and proliferation of the retinal pigment epithelium in cat eyes injected with tPA doses ≥ 50 *μ*g per 0.1 mL [23]. Hesse et al. reported large, inferior, exudative retinal detachment in four of five patients treated with tPA 100 *μ*g, which they considered to be an adverse effect [24, 25]. Hassan et al. did not find any evidence of retinal or other intraocular adverse effects in 15 patients who received intravitreal tPA doses of 25 *μ*g to 100 *μ*g per 0.1 mL [18]. Chen et al. described perturbations of the retinal pigment epithelium, poor recovery of visual acuity, and decreased photopic and scotopic A-waves and B-waves on the electroretinogram in a patient who received two 50 *μ*g injections of tPA within 3 days [26].

Hassan et al., Hrach et al., and Chen et al. recommended avoiding use of intravitreal tPA at concentrations > 25 *μ*g per 0.1 mL [18, 23, 26]. van Zeeburg and van Meurs concluded that 25 *μ*g per 0.1 mL is a safe and effective concentration of tPA for intravitreal use [27].

There has been some doubt with regard to whether intravitreal tPA is able to penetrate into the subretinal space in sufficient quantities to induce clot liquefaction [16]. Kamei et al. reported that intravitreal tPA labeled with fluorescein isothiocyanate did not diffuse across the neurosensory retina in the rabbit [14, 28]. However, albumin, a protein similar in molecular weight to tPA (68 kDa and 70 kDa, resp.), has been shown to diffuse into the subretinal space in less than one hour after intravitreal injection in rabbit eyes [29]. Coll et al. reported that intravitreal injection of tPA can penetrate the subretinal space via the intact rabbit retina and promote lysis of blood clots that have been present under the retina for 24 hours [18, 30]. In a pig model, Boone et al. demonstrated that subretinal clots were partially liquefied 24 hours after administration of intravitreal tPA [18, 31]. Some researchers have concluded that retinal microlesions develop because of stretching of the retina during acute bleeding and allow tPA to pass into the subretinal space [24, 25, 28, 32].

Heras-Mulero et al. reported that posterior vitrectomy and intravitreal administration of tPA (50 *μ*g per 0.1 mL) and SF6 gas were effective for the treatment of traumatic submacular hemorrhage in a 25-year-old patient [4]. Further, Doi et al. reported that vitrectomy in combination with subretinal injection of tPA (6.9 *μ*g per 0.1 mL) and air tamponade was a successful treatment for traumatic submacular hemorrhage in a 13-year-old boy [33].

Goldman et al. reported that pneumatic displacement using SF6 gas improved visual acuity in a 25-year-old patient with traumatic submacular hemorrhage [1]. Holland and Wiechens reported that pneumatic displacement using tPA (50 *μ*g per 0.1 mL) and SF6 gas was an effective treatment for traumatic submacular hemorrhage in a 37-year-old man and that pneumatic displacement using SF6 gas was effective for treatment of traumatic submacular hemorrhage in a 21-year-old male patient [2].

Our case was a 10-year-old boy with traumatic submacular hemorrhage. Injection of gas into an eye without posterior vitreous detachment may cause serious complications, such as vitreous hemorrhage and rhegmatogenous retinal detachment. Vitrectomy for a pediatric patient can be associated with serious complications, including rhegmatogenous retinal detachment and proliferative vitreoretinopathy [33]. Pneumatic displacement is less invasive than vitrectomy, so we chose to avoid vitrectomy and use injection of tPA (12.5 *μ*g per 0.05 mL) and SF6 gas in this patient. Vision was restored in our patient without the above-mentioned complications.

In conclusion, pneumatic displacement is an effective treatment for traumatic submacular hemorrhage, even in young patients. However, this is a single case report, and a prospective randomized controlled trial in a larger number of patients would be needed to assess the benefit of this technique in the management of pediatric patients with traumatic submacular hemorrhage.
